# Importance of cysteine residues in the C-terminal region of PGRL1 for PSI photoprotection in *Chlamydomonas reinhardtii*

**DOI:** 10.1093/plphys/kiag164

**Published:** 2026-03-24

**Authors:** Hiroko Takahashi, Yuki Okegawa, Atsuko Isu, Tetsuto Sato, Keisuke Yoshida, Ken-ichi Wakabayashi, Toru Hisabori, Yoshitaka Nishiyama

**Affiliations:** Graduate School of Science and Engineering, Saitama University, 255 Shimo-Okubo, Sakura-ku, Saitama 338-3570, Japan; Department of Molecular Biology and Biochemistry, Faculty of Science, Saitama University, 255 Shimo-Okubo, Sakura-ku, Saitama 338-3570, Japan; Institute of Plant and Science and Resources, Okayama University, Chuo 2-20-1, Kurashiki 710-0046, Japan; Laboratory for Chemistry and Life Science, Institute of Integrated Research, Institute of Science Tokyo, 4259 Nagatsuda-cho, Midori-ku, Yokohama 226-8501, Japan; Department of Molecular Biology and Biochemistry, Faculty of Science, Saitama University, 255 Shimo-Okubo, Sakura-ku, Saitama 338-3570, Japan; Laboratory for Chemistry and Life Science, Institute of Integrated Research, Institute of Science Tokyo, 4259 Nagatsuda-cho, Midori-ku, Yokohama 226-8501, Japan; Laboratory for Chemistry and Life Science, Institute of Integrated Research, Institute of Science Tokyo, 4259 Nagatsuda-cho, Midori-ku, Yokohama 226-8501, Japan; Faculty of Life Sciences, Kyoto Sangyo University, Motoyama, Kamigamo, Kita-ku, Kyoto 603-8555, Japan; Laboratory for Chemistry and Life Science, Institute of Integrated Research, Institute of Science Tokyo, 4259 Nagatsuda-cho, Midori-ku, Yokohama 226-8501, Japan; The Graduate University for Advanced Studies, SOKENDAI, Shonan Village, Hayama 240-0193, Japan; Graduate School of Science and Engineering, Saitama University, 255 Shimo-Okubo, Sakura-ku, Saitama 338-3570, Japan; Department of Molecular Biology and Biochemistry, Faculty of Science, Saitama University, 255 Shimo-Okubo, Sakura-ku, Saitama 338-3570, Japan

## Abstract

Photoinhibition is more pronounced in photosystem II (PSII) than in PSI. However, PSI is sensitive to photoinhibition in mutants of PROTON GRADIENT REGULATION5 (PGR5) and PGR5-LIKE1 (PGRL1) in both *Arabidopsis thaliana* and *Chlamydomonas reinhardtii*. In this study, we observed severe PSI photoinhibition in the PGRL1-deficient mutant (*pgrl1*) of *C. reinhardtii* under high light for 1 h. Because PSI photoinhibition was mitigated in *pgrl1*-complemented strains, PGRL1 was thought to play a crucial role in PSI photoprotection. To elucidate the mechanism of PGRL1-dependent PSI photoprotection, we introduced cysteine-to-serine (CS) substitutions at the 6 widely conserved cysteine residues (C63, C167, C256, C259, C284, and C287) of PGRL1 in *C. reinhardtii*. Among the transformants obtained, no CS variants at C256 and C259 accumulated PGRL1. The C63SC167S variant restored the PSI photosensitive phenotype, whereas the CS variants of the C-terminal region (C284SC287S, C284S, and C287S) exhibited PSI photoinhibition similar to *pgrl1* and destabilization of PGRL1 under high light. These variants failed to accumulate PGR5 even under growth light. These results suggest that CS substitutions at the C-terminal region affect the proper conformation of PGRL1, leading to its destabilization and disruption of the PGRL1–PGR5 interaction. Based on these findings, we conclude that PGRL1 C284 and C287 play important roles in regulating cyclic electron flow in *C. reinhardtii,* and consequently, in PSI photoprotection, by stabilizing PGRL1 and enabling PGR5 accumulation.

## Teaser

Proper conformation of the C-terminal region of PROTON GRADIENT REGULATION5 (PGR5)-LIKE1 is essential for PGR5 accumulation, which mediates photosystem I photoprotection in *Chlamydomonas reinhardtii*.

## Introduction

Inactivation of photosystem II (PSII) under high-light stress is referred to as photoinhibition (reviewed in Nishiyama and Murata 2014). PSI is relatively resistant to high-light illumination, except under chilling (reviewed in Sonoike 2011; 2025). Cyclic electron flow (CEF) around PSI is an alternative electron transport pathway in which PSI-derived electrons are transported to the plastoquinone pool, the cytochrome *b*_6_*f* complex (Cyt*b*_6_*f*), and PSI. A proton gradient across the thylakoid membrane formed by CEF induces photosynthetic control at the Cyt*b*_6_*f* complex, as well as nonphotochemical quenching (Nawrocki et al. 2019; Shikanai 2024). CEF contributes to PSII photoprotection by inducing nonphotochemical quenching. In addition, CEF protects PSI by suppressing electron influx into PSI on the donor side and by mitigating over-reduction on the acceptor side (Shikanai 2024). Proton gradient regulation 5 (PGR5) and PGR5-like photosynthetic phenotype 1 (PGRL1) mediate the CEF pathway. Hereafter, this pathway is referred to as the PGR-dependent pathway (Shikanai 2014). PGR5 and PGRL1 are thylakoid membrane proteins with approximate molecular masses of 10 and 30 kDa, respectively (Munekage et al. 2002; DalCorso et al. 2008). In mutants deficient in PGR5 (*pgr5*) or PGRL1 (*pgrl1*), PSI is inactivated under fluctuating-light and high-light conditions in both *Arabidopsis thaliana* (Munekage et al. 2002; DalCorso et al. 2008; Suorsa et al. 2012) and *Chlamydomonas reinhardtii* (Johnson et al. 2014; Bergner et al. 2015). PGR5 does not accumulate in *pgrl1*, indicating that PGRL1 is required for stable PGR5 accumulation (DalCorso et al. 2008; Johnson et al. 2014).

In *A. thaliana*, PGRL1 and PGR5 function as a heterodimer to mediate the PGR-dependent pathway of CEF (DalCorso et al. 2008; Hertle et al. 2013). Ruhle et al. reported that PGRL2, a homolog of PGRL1 found only in land plants, destabilizes PGR5 to prevent its overaccumulation (Ruhle et al. 2021). The activity of the PGR-dependent pathway is regulated by thioredoxin (Trx) *m4* through a redox-dependent interaction with a cysteine residue at the N-terminal region of PGRL1 (Okegawa and Motohashi 2020).

Under over-reducing conditions, formation of a supercomplex (CEF complex), containing PSI, Cyt*b*_6_*f*, Ferredoxin-NADP^+^ oxidoreductase (FNR), PGRL1, and 2 Chlorophyceae-specific proteins (ANR1 and PETO), promotes PGR-dependent CEF in *C. reinhardtii* (Iwai et al. 2010; Terashima et al. 2012; Takahashi et al. 2013, 2016). Although PGR5 has not been identified as a component of the CEF complex, Petroutsos et al. proposed that PGRL1 and PGR5 are coregulated (Petroutsos et al. 2009). Furthermore, *pgr5* did not accelerate CEF rate under over-reducing conditions (Johnson et al. 2014), indicating that in *C. reinhardtii,* PGR5 plays a role in CEF with PGRL1. Mosebach et al. reported that the amount of thylakoid membrane-bound FNR decreased in *pgr5* and *pgrl1,* especially in the *pgr5pgrl1* double mutant (Mosebach et al. 2017). They proposed that membrane-bound FNR recruited by PGR5/PGRL1 facilitates electron transfer from PSI to the CEF pathway through ferredoxin 1. Recently, FNR tethering to PSI was reported to enhance CEF activity (Emrich-Mills et al. 2025). Therefore, membrane-bound FNR may be crucial for the PGR-dependent CEF pathway. In contrast to *A. thaliana*, studies are lacking on Trx-mediated regulation of the CEF pathway in *C. reinhardtii*.

PGRL1 possesses 2 transmembrane domains and 6 cysteine residues conserved from green algae to land plants (DalCorso et al. 2008). Two of the cysteine residues are in the N-terminal region, whereas the others are in the C-terminal region. Hertle et al. characterized the recombinant *A. thaliana* PGRL1 protein (*At*PGRL1) with cysteine-to-serine (CS) substitutions and predicted the function of each cysteine residue (Hertle et al. 2013). Two cysteine residues in the N-terminal region, C22 and C123, were predicted to serve as the binding site for Trx. This is consistent with in vivo studies on cysteine residues in the N-terminal region of *At*PGRL1 (Okegawa and Motohashi 2020; Wolf et al. 2020). Each of the 2 pairs of C-terminal cysteine residues (C212 and C215 as well as C240 and C243) forms a CXXC motif that is involved in binding iron-sulfur clusters and iron or zinc atoms, as well as forming disulfide bonds (Belmonte and Mansy 2017). Structural predictions of *At*PGRL1 suggested that these 4 cysteine residues serve as ligands for a zinc atom (Chaturvedi et al. 2024; Walz et al. 2025). Hertle et al. proposed that residues C212 and C215 form the binding site for iron-containing cofactors, whereas residues C240 and C243 function as the binding site for PGR5 (Hertle et al. 2013). However, in vivo studies on the roles of the cysteine residues in the C-terminal region of PGRL1 are lacking in *A. thaliana* and *C. reinhardtii*.

In this study, we characterized PGRL1-CS-substituted strains to elucidate how the cysteine residues of the *C. reinhardtii* PGRL1 protein (*Cr*PGRL1) contribute to PSI photoprotection. Here, these cysteine residues are numbered C63, C167, C256, C259, C284, and C287. The N-terminal CS variant (C63SC167S), which allows PGR5 accumulation, was tolerant to PSI photoinhibition, whereas the C-terminal CS variants (C284SC287S, C284S, and C287S), lacking PGR5 accumulation, were sensitive. In these variants, PGRL1 was unstable under high light. Our structural modeling of *Cr*PGRL1 using AlphaFold3 suggests that C284 and C287 coordinate a zinc atom with the other 2 cysteine residues and that PGR5 interacts with PGRL1 at its C-terminal region. These results suggest that C284 and C287 play crucial roles in maintaining the conformation of PGRL1, stabilizing the protein and enabling PGR5 accumulation.

## Materials and methods

### Strains and growth conditions

The WT is the strain CC-124. Dr. Giles Peltier kindly provided the null-mutant of PGRL1 (*pgrl1*) used as the recipient strain for site-directed mutagenesis (Tolleter et al. 2011). In this study, *pgrl1* transformed with EV was used for all analyses. WT strain T222+, which is the recipient strain for the mutagenesis in which the PGR5-deficient mutant (*pgr5*) was obtained by Dent et al. (2005), was kindly provided by Dr. Shin-Ichiro Ozawa (Okayama University, Japan). The *pgr5* mutant and its complemented strain (*pgr5* + PGR5) were kindly provided by Dr. Xenie Johnson (Johnson et al. 2014). Cells were cultured in Tris-acetate-phosphate (TAP) medium (Gorman and Levine 1965) at 25 °C under 80 μmol photons m ^−2^ s^−1^ with shaking (90 to 100 rpm), unless described otherwise. Cells grown to midlog phase (2 to 5 × 10^6^ cells mL^−1^) were used for the experiments. For photoautotrophic growth, high-salt minimal medium (Sueoka 1960) was used.

### Construction to generate cysteine-serine substituted mutants

The coding sequence of PGRL1 in *C. reinhardtii* (Cre07.g340200, hereafter CrPGRL1) was amplified by PCR with primers, CrPGRL1OHpET21b_Fw and CrPGRL1OHpET21b_Rv (Table S2). The amplified fragment was cloned into pET21b to add a His-tag to the C-terminus by Gibson assembly. The resulting plasmid (CrPGRL1 coding sequence with 8-His tag) was subcloned into pSL18-pHygromycine (Hyg) 3, kindly provided by Dr. Hiroshi Kuroda (Nellaepalli et al. 2018), through Gibson assembly using the primers CrPGRL1cdsOHpSXY2007_Fw and CrPGRL1cdsOHpSXY2007_Rv (Table S2). The CS substitution (TCC to TGC) was introduced by PCR. Prior to introduction into *C. reinhardtii,* the vector was linearized by digestion with KpnI and ScaI. *C. reinhardtii* was transformed with PGRL1 as described by Yamano et al. (2013). Transformants were selected on a TAP-agarose plate containing 10 μg mL^−1^ Hyg. Hyg-resistant transformants were subjected to PCR to amplify the full-length of the coding sequence using the primers, proPSAD and terPSAD (Table S2). To check the level of PGRL1, the clones introducing *Cr*PGRL1 were analyzed through immunoblotting.

### Measurement of PSI activity

Measurement of PSI activity was monitored by measuring the photooxidation of P700 using a Joliot type spectrophotometer-10 (JTS-10, Biologic, France). *C. reinhardtii* cells were collected by centrifugation at 830 × *g*, 10 min, 25 °C, and resuspended in 20 mM HEPES-NaOH pH 7.5 containing 10% Ficoll. The concentration of P700 (ΔAbs. 705 nm) was adjusted among the variants to approximately −3 × 10^3^. In this case, the cell concentration was 2 to 3 × 10^6^ cells mL^−1^ among the strains. The cell suspension was illuminated with high light (1,400 μmol photons m^−2^ s^−1^) for 1 h. Photooxidation of P700 was measured as described in Alric et al. (2010), using the actinic light (150 μmol photons m^−2^ s^−1^) in the presence of 10 μM 3-(3′,4′-dichlorophenyl)-1,1-dimethylurea and with a pulse of 25,000 μmol photons m^−2^ s^−1^ for 100 ms to determine the maximum level of P700. The relative activity of PSI was determined based on the level of photooxidizable P700 at 0 min of treatment as 100% of PSI activity.

### SDS-PAGE and immunoblotting analysis

*Chlamydomonas* cells were harvested by centrifugation at 830 × *g*, 10 min, 4 °C, to analyze total cellular protein. The pellets were suspended in 0.1 M DTT, 0.1 M Na_2_CO_3_, and the 1 × protease inhibitor cocktail and solubilized with 2% SDS. The preparations were heated at 95 °C for 1 min and centrifuged at 20,000 × *g*, 5 min, 4 °C. The chlorophyll concentration of the supernatants was determined as described (Porra et al. 1989). The supernatants were subjected to SDS–PAGE (Laemmli 1970) with equal amounts of chlorophyll per lane. To detect *Cr*PGR5, we used a commercial precast gel, p-PAGEL (ATTO, Japan), with running buffers of the Tris-tricine system (Schagger and von Jagow 1987). For immunoblotting, proteins were electrophoretically transferred to a polyvinylidene difluoride membrane (Clear Blot P+ membrane, ATTO). ATP-γ, PGRL1, and PSAD were detected with the corresponding polyclonal antibodies conjugated to horseradish peroxidase (GE Healthcare). The signals were detected using enhanced chemiluminescence reagent (ImmunoStar, Fujifilm Wako Pure Chemical Corporation) and C-DiGit blot scanner (LI-COR) or Luminograph I (ATTO). Signal intensity was quantified using Image Gauge version 4.0 software (Fujifilm).

### Redox assay

Cell concentration was adjusted to 2 to 3 × 10^6^ cells mL^−1^. Cell suspensions were incubated in the dark for 1 h. After sampling the dark-adapted cells was completed, the cells were illuminated at 1,400 μmol photons m^−2^ s^−1^ for 40 min and sampled as illuminated cells. The sampled cells were immediately fixed with 10% TCA and frozen in liquid nitrogen. The frozen cell suspensions were thawed by centrifugation at 20,000 × *g*, 5 to 10 min, 20 °C. The resulting pellets were washed twice with cold acetone. The pellets were solubilized with 2% SDS, 62.5 mM Tris–HCl (pH 6.8), 10% glycerol, 0.01% bromophenol blue, and 1× protease inhibitor cocktail, with or without 4 mM 4-acetamido-4′-maleimidylstilbene-2,2′-disulfonic acid (AMS, Thermo Fischer Scientific). Samples were incubated at room temperature for 1 h in the dark and heated at 70 °C for 15 min. Protein concentration was determined with the BCA method using the Protein Assay BCA Kit (Nacalai Tesque). The samples were subjected to nonreducing SDS–PAGE and immunoblotting.

### Protein stability assay

Cell concentration was adjusted to 2 to 3 × 10^6^ cells mL^−1^. The cell suspensions were incubated in the presence or absence of 10 μg mL^−1^ cycloheximide under growth light (in the growth chamber) or high light (1,400 μmol photons m^−2^ s^−1^) for 1 h. Cell suspensions under high light were replaced in the growth chamber and incubated for 1 h. Cells were collected at 0, 1, and 2 h by centrifugation at 20,000 × *g*, 10 min, 4 °C. The cells were resuspended with 0.1 M DTT, 0.1 M Na_2_CO_3_, and 1× protease inhibitor cocktail and frozen in liquid N_2_. The following procedures were performed as described in the *SDS–PAGE and immunoblotting analysis* section.

### Generation of the CrPGR5-antibody

The preparation of the antigen to generate an antiserum against the *Cr*PGR5 protein was principally based on the method of Okegawa and Motohashi (2020). A four-tandem repeat of the predicted mature sequence of CrPGR5 (residue 53 to 141), synthesized by Eurofin Genomics, was cloned into the pET21 vector. The expression of *Cr*PGR5 in *Escherichia coli* BL21 (DE3) strain was induced with 0.5 mM isopropyl β-D-thiogalactopyranoside at OD_600_ of 0.4 to 0.5. The *E. coli* cells were cultured for 3 h at 37 °C, and harvested by centrifugation (3,005 × *g*, 10 min, 4 °C). The pellet was resuspended in lysis buffer (25 mM Tris–HCl [pH 7.5], 150 mM NaCl, and 2 mM EDTA) and disrupted by sonication. The recombinant *Cr*PGR5 repeat protein, present in inclusion bodies, was washed with buffer containing 0.2% Triton-X 100 and further washed with buffer containing 8 M urea. The washed fraction was solubilized with buffer containing 6 M guanidium chloride and centrifuged at 11,000 × *g*, 10 min, 4 °C. The supernatant was used to immunize rabbits.

## Results

### Complementation of the pgrl1 mutation with PGRL1 restored PSI photoinhibition

**Figure 1:**
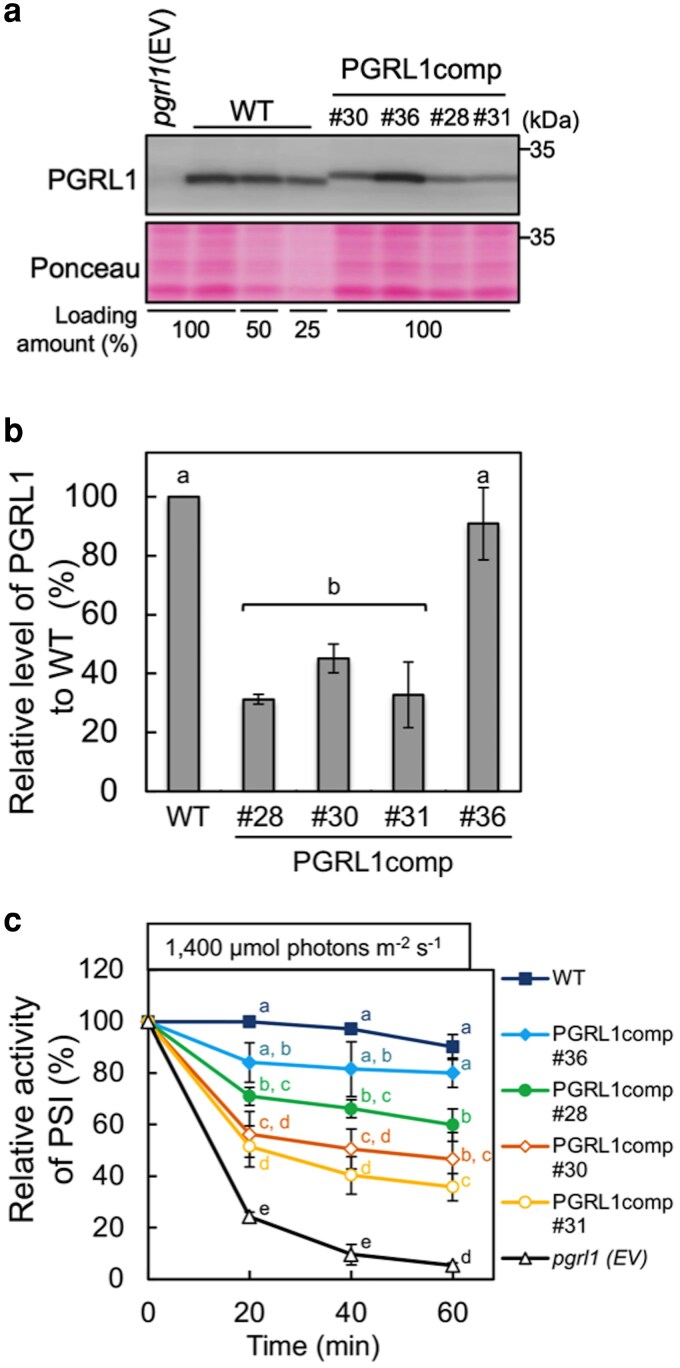
PGRL1 level and PSI activity in PGRL1-complemented strains. **(a)** Total cellular protein (equivalent to 1 μg Chl as 100%) extracted from the wild type (WT), *pgrl1* (transformed with the empty vector [EV]), and the PGRL1-complemented strains (PGRL1comp#28, -#30, -#31, and -#36) were immunoblotted using the PGRL1 antiserum. Ponceau-stained protein profiles are shown as loading controls. **(b)** The level of PGRL1 relative to that in WT was quantified by analyzing signal intensity in the PGRL1comp strains. Values represent means of three biological replicates; error bars indicate ± SD. Different letters indicate the statistically significant differences, as determined by Tukey's test (*P* < 0.05). **(c)** PSI activity in the WT, *pgrl1* (EV), and PGRL1comp strains under 1,400 μmol photons m^−2^ s^−1^. PSI activity was determined based on the maximum level of photooxidizable P700 (see Materials and Methods). The symbols represent the strains as follows: filled square, WT; filled diamond, PGRL1comp#36; filled circle, PGRL1comp#28; open diamond, PGRL1comp#30; open circle, PGRL1comp#31; and open triangle, *pgrl1.* Values represent means of 3 biological replicates; error bars indicate ± SD. Different letters indicate statistically significant differences among the strains at each time point as determined by Tukey's test (*P* < 0.05).

The *pgrl1* mutant of *C. reinhardtii* exhibited lower nonphotochemical quenching than the wild type (WT) (Tolleter et al. 2011). PSI accumulation did not change after 6 h of exposure to 200 μmol photons m^−2^ s^−1^ of light (Kukuczka et al. 2014), whereas P700 photooxidation was partially inhibited after 4 h of exposure to the same light intensity (Bergner et al. 2015). In this study, we analyzed PSI activity in the WT and *pgrl1* mutant for 1 h under high light (1,400 μmol photons m^−2^ s^−1^) to monitor how short-term high-light treatment affects PSI activity. Figure S1 shows the kinetics of P700 photooxidation measured in the presence of 3-(3′,4′-dichlorophenyl)-1,1-dimethylurea in the WT and *pgrl1* mutant of *C. reinhardtii* during this treatment. To determine the maximum oxidation level of P700, a pulse illumination was applied at the end of actinic light illumination. In WT, the maximum oxidation level was slightly lower after 1 h of high-light treatment (Figure S1a), indicating that PSI activity is largely maintained under high light. Similar to the WT, P700 was photooxidized in *pgrl1* before high-light treatment (Figure S1b), indicating that PSI is functional under growth conditions in *pgrl1*. However, after high-light treatment, P700 was hardly photooxidized (Figure S1b). This suppression was not alleviated by the addition of 1 mM methylviologen—an artificial electron acceptor that relieves PSI acceptor-side over-reduction—indicating that suppression of P700 photooxidation is attributable to impaired electron transfer within PSI, namely, PSI photoinhibition. This result indicates that PGRL1 is required for the maintenance of a functional PSI under high light. We generated complemented strains of *pgrl1* by introducing a *Cr*PGRL1-coding sequence fused with a His-tag at its C-terminus. Figure 1, a and b shows the level of PGRL1 accumulation in the complemented strains (PGRL1comp). The PGRL1 band in PGRL1comp was slightly upshifted due to the attached His-tag. The amount of PGRL1 in the PGRL1comp#28, -#30, and -#31 strains was 30% to 40% of that in WT, whereas the amount in the PGRL1comp#36 strain was comparable to that in WT (Fig. 1, a and b). The cells of WT, *pgrl1* (transformed with the empty vector, EV), and the PGRL1comp strains were treated with high light (Fig. 1c). PSI activity in the PGRL1comp#36 strain was maintained at ∼80% of the initial activity, whereas in the PGRL1comp#28, -#30, and -#31 strains, activity dropped to 40% to 60% of the initial activity at the onset of the illumination. These results suggest that a certain level of PGRL1 accumulation is necessary to maintain PSI activity under high light. Because each PGRL1comp strain exhibited different levels of PGRL1 accumulation, we primarily used the PGRL1comp#30 and -#36 strains as a less-PGRL1 strain and a WT-level PGRL1 strain for analysis.

### Serine-substitution(s) in C284, C287, or C284 and C287 did not complement the phenotype of PSI photoinhibition

**Figure 2:**
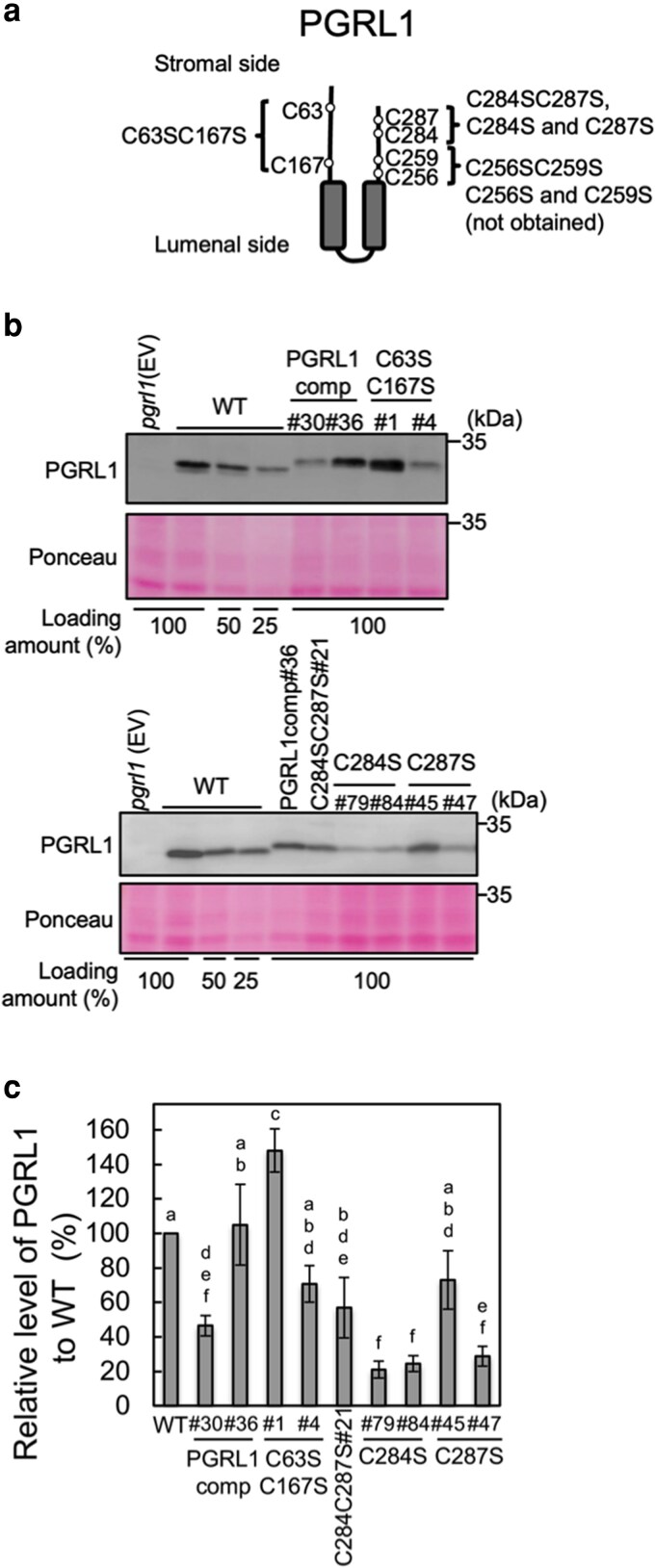
Generation of PGRL1 cysteine-to-serine substituted mutants. **(a)** Schematic representation of the PGRL1 protein, showing the positions of cysteine-to-serine (CS) substitutions as open circles and 2 transmembrane helices as gray boxes. **(b)** Total cellular protein (equivalent to 1 μg Chl as 100%) extracted from the PGRL1 variants with CS substitutions was immunoblotted using the PGRL1 antiserum. Ponceau-stained protein profiles are shown as loading controls. **(c)** The level of PGRL1 relative to that in WT was quantified by analyzing signal intensity in variants with CS substitutions. Values represent means of 3 biological replicates; error bars indicate ± SD. Different letters indicate the statistically significant differences among the strains as determined by Tukey's test (*P* < 0.05).

**Figure 3:**
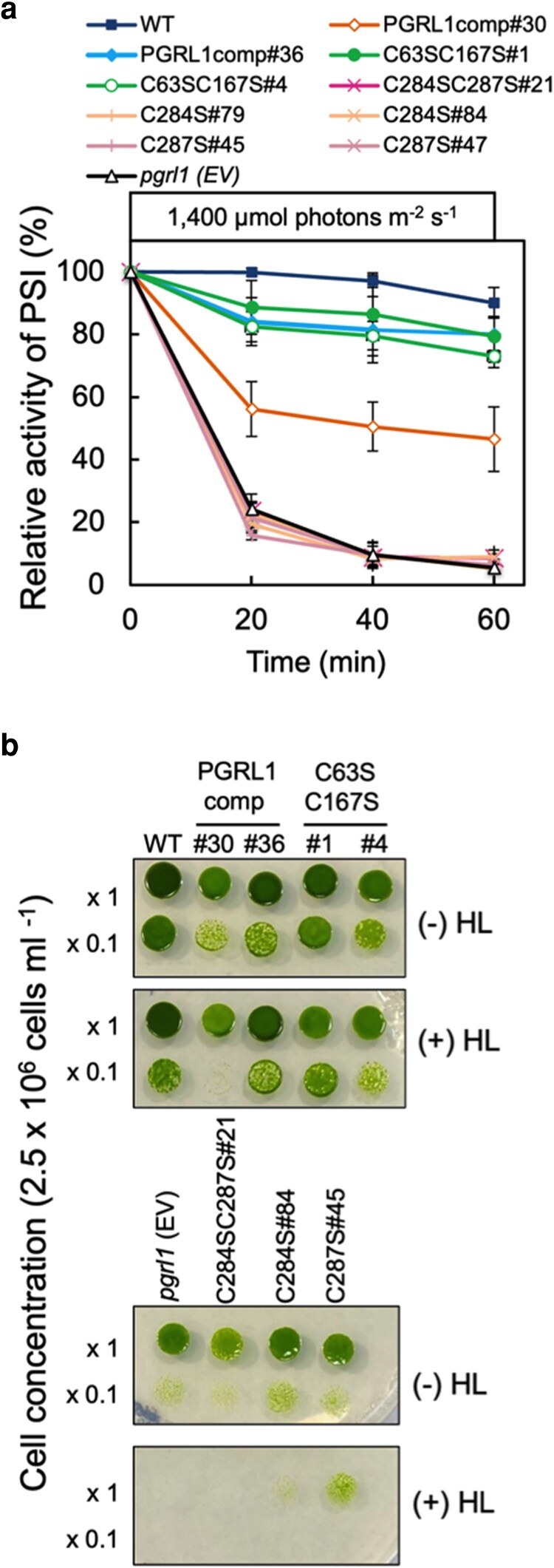
PSI activity under high light and cell growth in PGRL1 CS variants. **(a)** WT, *pgrl1* (EV), and CS variant cells were illuminated with high light (1,400 μmol photons m^−2^ s^−1^). PSI activity was determined as shown in Fig. 1c. The symbols represent the strains as follows: the filled square, WT; filled circle, C63SC167S#1; filled diamond, PGRL1comp#36; open circle, C63SC167S#4; open diamond, PGRL1comp#30; cross, C284SC287S; times sign and cross, C284S#79 and C284S#84; times sign and cross, C287S#45 and C287S#47; and open triangle, *pgrl1.* Values represent means of 3 biological replicates; error bars indicate ± SD. **(b)** Cell growth of WT, *pgrl1* (EV), PGRL1comp strains (#30 and #36), and CS variants on Tris-acetate-phosphate (TAP) agar plates with or without high light treatment. Cells on TAP agar plates were illuminated at 1,400 μmol photons m^−2^ s^−1^ for 1 h and kept under the growth light for 3 d, represented as (+) HL. As a control, the cells on the TAP plate were kept under the growth light without high light treatment for 3 d, represented as (−) HL.

We aimed to generate the complemented strains of *pgrl1* carrying substitutions of each cysteine pair in the N-terminal (C63 and C167) or C-terminal (C256 and C259 or C284 and C287) region of PGRL1 with serine (Fig. 2a). Furthermore, we introduced every single substitution at C256, C259, C284, and C287. Among the 5 clones of the C63SC167S transformants, 4 clones accumulated PGRL1 at 60% to 150% of the WT level (Table S1). We then used C63SC167S#1 as the overexpressing variant and -#4 as the low-expressing variant for analysis (Fig. 2, b and c). We could not obtain any transformant clones that accumulated PGRL1 for the C256S, C259S, and C256SC259S variants (Table S1). Among the 32 clones of the C284SC287S transformants, one clone, designated C284SC287S#21, accumulated PGRL1 at ∼60% of the WT level (Table S1; Fig. 2, b and c). In addition, among the 64 transformants tested, C287S#45 accumulated PGRL1 at ∼60% of the WT level (Fig. 2, b and c). Other transformants, C284S (#79, #84, and #85) and C287S (#47 and #52), accumulated PGRL1 at 20% to 30% of the WT level (Table S1; Fig. 2, b, c, and Figure S2a). We primarily analyzed C284SC287S#21 as the double C-terminal CS-substituted variant, as well as C284S#84 and C287S#45 as the single corresponding variants, respectively, for analysis. Accumulation of *PGRL1* transcripts in C284SC287S#21 (hereafter C284SC287S), C284S#84 (hereafter C284S), and C287S#45 (hereafter C287S) cells was detected using RT-qPCR. Transcriptional levels in C284SC287S and C287S were comparable with those in WT (Figure S3). The accumulation of PSI protein was unaffected in the C284SC287S, C284S, and C287S variants, as well as in *pgrl1* (Figure S2a). To examine the light sensitivity of PSI in the CS variants, PSI activity was measured under high light (Fig. 3a). In strains C63SC167S#1 and -#4, PSI activity during illumination was maintained at 70% to 80% of the initial activity, indicating restoration of photosensitivity (Fig. 3a). P700 was normally photooxidized before the treatment in the C284SC287S, C284S, and C287S variants, as observed in the PGRL1comp#36 strain (Figure S2, b–e). Nevertheless, these variants lost PSI activity under high light, dropping to ∼20% and 10% of the initial levels after 20 and 60 min of illumination, respectively (Fig. 3a). These results were similar to those for *pgrl1*, suggesting that PGRL1 C284 and C287 are important for maintaining PSI activity under high light. Cell growth in the CS variants was assessed under growth light (80 μmol photons m^−2^ s^−1^) with and without prior high-light treatment (Fig. 3b). The growth of the PGRL1comp and C63SC167S variants was comparable to that of WT under both conditions, excluding the PGRL1comp#30 strain, which showed slightly slower growth after high-light exposure (Fig. 3b, upper panel). By contrast, the growth of the C284SC287S, C284S, and C287S variants was impaired even under growth-light conditions and was further suppressed by high-light treatment (Fig. 3b, lower panel).

### All CS variants failed to form the high molecular weight (HMW) PGRL1 complexes

*Cr*PGRL1 was oxidized by 100 mM hydrogen peroxide and reduced by 100 mM dithiothreitol (Petroutsos et al. 2009). However, studies are lacking on the redox profile of PGRL1 upon exposure to high light in *C. reinhardtii*. In this study, total cellular protein was prepared from dark-adapted and illuminated cells grown under mixotrophic conditions. The redox profiles of PGRL1 were analyzed by immunoblotting after labeling the free thiol groups. Figure S4, a and b show the redox profiles of the CF_1_-γ subunit as a positive control of the assay and PGRL1 under dark and light conditions, respectively. The CF_1_-γ subunit showed the upshift of the band under light conditions due to photoreduction (Yoshida et al. 2014). The redox profile of PGRL1 in WT revealed two HMW bands (indicated by asterisks in Figure S4b). The apparent sizes of the upper and lower bands seemed to be close to those of the PGRL1 homodimer and PGRL1–Trx *m* complex, respectively, as reported in *A. thaliana* (Okegawa and Motohashi 2020). After exposure to high light, the reduced form of the PGRL1 monomer was detected in addition to the two HMW bands (Figure S4b, arrowhead). The same assay was performed in the PGRL1comp and CS variants (Figure S4c). The result obtained in the PGRL1comp#36 strain was principally the same as that in WT. However, for any CS variant, the HMW bands were not detected; only the monomeric form was detected both under dark and light conditions (Figure S4c left panel). Under reducing conditions, the HMW bands disappeared; only monomeric PGRL1 was detected in all variants (Figure S4c right panel). Thus, the protein complexes corresponding to HMW bands seemed to be formed in a redox-dependent manner.

### Serine-substitution(s) in C284, C287, or C284 and C287 destabilized PGRL1 under high light

**Figure 4:**
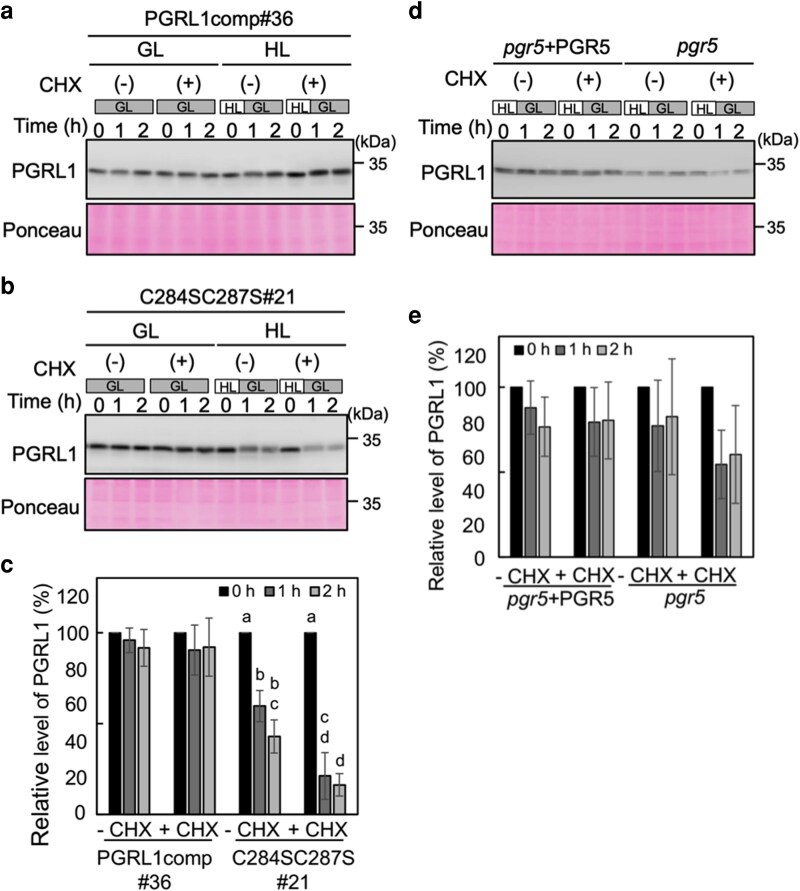
Stability of PGRL1 under high light. PGRL1comp#36 **(a)** and C284SC287S **(b)** were incubated for 1 h under the growth light at 80 μmol photons m^−2^ s^−1^ (GL) or high light at 1,400 μmol photons m^−2^ s^−1^ (HL) in the absence or presence of 10 μg mL^−1^ cycloheximide (CHX), followed by incubation under growth light for 1 h. Cells were harvested at the indicated time points and immunoblotted using the PGRL1 antiserum. In total, 0.5 μg of Chl *a* + *b* was loaded in each lane. Ponceau-stained protein profiles are shown as loading controls. **(c)** The levels of PGRL1 under high light relative to the pretreatment level were quantified by analyzing signal intensity. Values represent means of 3 biological replicates; error bars indicate ± SD. Different letters indicate the statistically significant differences between the pretreatment levels and other conditions in C284SC287S#21, as determined by Tukey's test (*P* < 0.05). **(d)** The stability of PGRL1 was tested in *pgr5* and the *pgr5* + PGR5 strain, following the method used in Fig. 4a and b. **(e)** The levels of PGRL1 under high light relative to the pretreatment levels were quantified by analyzing signal intensity. Values represent means of three biological replicates; error bars indicate ± SD.

We compared the accumulation of PGRL1 between mixotrophically grown and photoautotrophically grown cells (Figure S5). Even under growth-light conditions, the absence of CO_2_ supplementation can induce the acceptor-side limitation of PSI due to the downregulation of CO_2_ fixation, mimicking a type of light-stress condition (Sonoike 2011; Lima-Melo et al. 2021). Compared to the WT, PGRL1 accumulation was much lower in the C284SC287S, C284S, and C287S variants under photoautotrophic conditions without CO_2_ supplementation than under mixotrophic conditions (Figure S5). This result suggests that PGRL1 is unstable under light stress. To assess the stability of PGRL1, the mixotrophic cells of the PGRL1comp#36 strain and C284SC287S were incubated in the absence or presence of cycloheximide under growth light or high light for 1 h, followed by 1 h under growth light (Fig. 4, a and b). Total cellular protein was analyzed by immunoblotting to quantify PGRL1. The levels of PGRL1 relative to pretreatment levels are shown in Fig. 4c. In the PGRL1comp#36 strain, PGRL1 level was unchanged under all conditions (Fig. 4, a and c). In WT and C63SC167S#1 cells, PGRL1 levels were maintained during and after high-light treatment (Figure S6a). Consistently, PGRL1 level was unchanged after high-light treatment in the PGRL1comp#30 strain, in which PGRL1 level was ∼45% of that of WT and showed partial PSI photoinhibition, as shown in Fig. 1 (Figure S6b). In the C284SC287S strain, PGRL1 levels were maintained during incubation under growth-light conditions, even in the presence of cycloheximide. However, PGRL1 levels in the C284SC287S variant decreased when the cells were incubated under high light (Fig. 4, b and c). This decrease was further enhanced in the presence of cycloheximide. Consistently, the C287S variant showed a decrease in PGRL1 levels under high light (Figure S6c). These results suggest that the substitutions of C284 and C287 destabilize PGRL1 under high light. Considering the role of PGR5 in PGRL1 stabilization, we examined PGRL1 stabilization in *pgr5* and the complemented strain (*pgr5* + PGR5) under high light (Fig. 4, d and e). Although PGRL1 accumulation appeared to decrease in *pgr5* after high-light treatment, the differences between pretreatment and posttreatment values were statistically nonsignificant. These results indicate that the CS substitutions at residues C284 and C287 in PGRL1 destabilize the protein under high light; the absence of PGR5 may not significantly impact PGRL1 stability.

### Serine-substitution(s) in C284, C287, or C284 and C287 prevented the PGR5 accumulation

**Figure 5:**
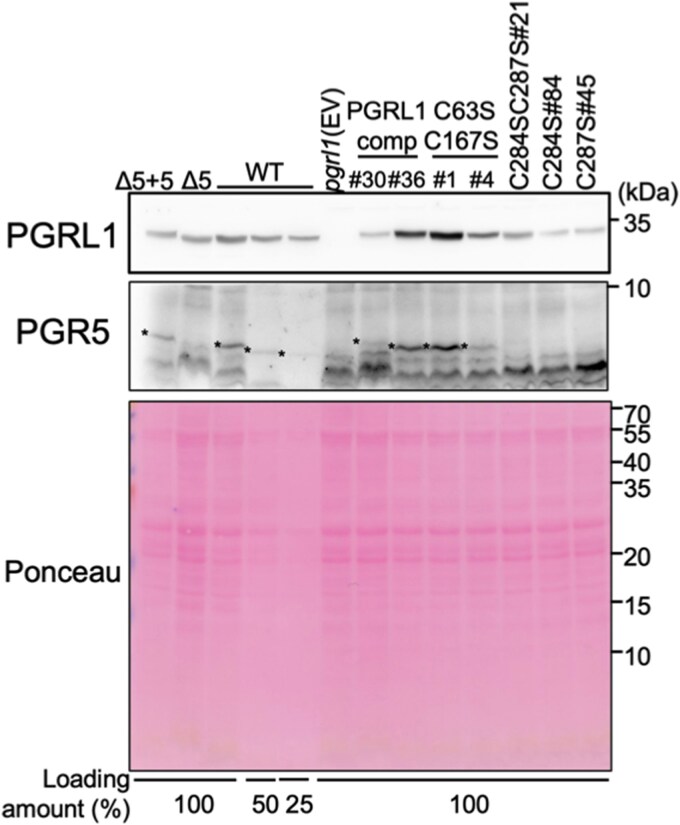
Accumulation of PGR5 in PGRL1-CS variants. Total cellular protein (equivalent to 1 μg Chl as 100%) was extracted from WT; *pgr5* (Δ5); *pgr5* + PGR5 (Δ5 + 5); *pgrl1* (EV); PGRL1comp strains (#30 and #36); and CS variants (C63SC167S#1 and -#4, C284SC287S#21, C284S#84, and C287S#45) and immunoblotted using PGRL1 and PGR5 antisera. Asterisk indicates the position of the PGR5 band. The results from the other 3 biological replicates are presented in Figure S7, b–d.

To analyze PGR5 accumulation in the CS variants, we generated an antiserum against *Cr*PGR5. Although several nonspecific cross-reactions were observed in the blot probed with the anti-*Cr*PGR5 serum, a major band absent in *pgr5* was detected in WT and *pgr5* + PGR5 strains (presented in the figures, as Δ5 + 5) (Figure S7a). Consistent with that in *A. thaliana and C. reinhardtii* (DalCorso et al. 2008; Johnson et al. 2014), PGR5 was not detected in *pgrl1* (Fig. 5; Figure S7, b–d). PGR5 was detected in the PGRL1comp strains and N-terminal CS variants; however, it was absent in C-terminal CS variants (Fig. 5; Figure S7, b–d), suggesting that CS substitutions in the C-terminal region prevented PGR5 accumulation. PSI activity in *pgr5* under high light decreased to ∼40% of the initial activity (Figure S8), indicating that PSI photoinhibition occurs in *pgr5*, as reported (Munekage et al. 2002; Johnson et al. 2014). This result supports the hypothesis that the PSI photoinhibition in C-terminal CS variants is caused by the absence of PGR5.

## Discussion

Over-reduction of the PSI acceptor side leads to the production of reactive oxygen species, which cause PSI photoinhibition by destroying the iron-sulfur clusters in PSI (Sonoike et al. 1995; Tiwari et al. 2016, 2024). Under high or fluctuating light conditions, increased electron input from PSII leads to the over-reduction of the PSI acceptor side, particularly in *pgr5* lacking CEF (Munekage et al. 2002; Suorsa et al. 2012; Johnson et al. 2014). Consistently, PSI photosensitivity in *pgr5* was partially alleviated by deleting FtsH—a protease that degrades damaged D1. This suggests that delayed PSII repair helps maintain the electron transport chain in a more oxidized state, thereby alleviating PSI photoinhibition (Ozawa et al. 2024).

Under high light, P700 photooxidation in *pgrl1* was readily inhibited, even after just 1 h of exposure. This PSI photoinhibition phenotype in *pgrl1* was restored in the complemented strains and C63SC167S variants in which PGRL1 is stable and PGR5 accumulates, as observed in WT (Figs. 3a, Fig. 5, Figure S6a, and Figure S7, b–d). This suggests that residues C63 and C167 are not involved in maintaining PSI activity under high light. By contrast, PSI activity in the C284SC287S, C284S, and C287S variants significantly decreased during high-light exposure (Fig. 3a). In these mutants, PGRL1 was highly unstable under high light, and PGR5 failed to accumulate even under growth-light conditions (Fig. 4, b–c, Fig. 5, Figure S6c, and Figure S7, b–d). These results support a model in which the accumulation of PGRL1 and PGR5 is required for CEF-dependent PSI photoprotection.

Cell growth in the PGRL1comp strains and N-terminal CS variants was comparable with that in WT, whereas growth in *pgrl1* and C-terminal CS variants was slower even under growth-light conditions (Fig. 3b). These findings suggest that growth condition (80 μmol photons m^−2^ s^−1^, without CO_2_ supplementation) induces PSI photoinhibition in cells spotted on an agar plate, particularly during the early growth phase. After exposure to high light for 1 h, cell growth was unaffected in the PGRL1comp#36 strain and C63SC167S variants and slightly delayed in the PGRL1comp#30 strain. This appears to be caused by the reduced accumulation of PGRL1 and PGR5 (Figs. 1a and 5). Cell growth was impaired in *pgrl1*, the C284SC284S, C284S, and C287S variants after exposure to high light. This suggests that once PSI photoinhibition occurs, cell growth is severely inhibited even under mixotrophic condition.

PSI photoinhibition appeared milder in *pgr5* than in *pgrl1* (Fig. 1c and Figure S8). However, a direct comparison is not possible due to differences in genetic backgrounds.

### Redox-dependent HMW complexes disappeared in CS variants

Redox assay of PGRL1 revealed 2 HMW PGRL1 bands in the WT and PGRL1comp#36 strain under dark (Figure S4). The apparent size of the upper and lower HMW bands corresponded with the size of the PGRL1 dimer and PGRL1–Trx *m* complex, respectively, as reported in *A. thaliana* (Okegawa and Motohashi 2020). We were unable to identify the band migrating just above 40 kDa. Although this band may correspond to the PGRL1–PGR5 complex, we do not have experimental evidence to support this hypothesis. The band of the PGRL1 monomer appeared after shifting to light. Based on the model proposed in a report (Okegawa and Motohashi 2020), CEF activity is inhibited by interacting with Trx *m4* or dimerization under dark conditions and at the steady state of photosynthesis. The PGRL1 monomer, reduced by electrons from photosynthetic electron flow, becomes active in mediating CEF during the induction of photosynthesis. Under high light, the necessity for CEF likely increased, which may have increased the monomeric form of PGRL1. The first and second cysteine residues of PGRL1 are involved in dimerization, and the second cysteine residue also serves as the binding site for Trx *m4* in *A. thaliana* (Okegawa and Motohashi 2020). In an in vitro study of *At*PGRL1, Hertle et al. showed that CS substitutions in the C-terminal region did not affect dimerization. However, Wolf et al. characterized the N- and C-terminal cysteine–alanine substituted mutants in vivo and showed that 2 disulfide-bridged complexes were not detected in these mutants of *A. thaliana*, supporting the conclusion that all cysteine residues of PGRL1 are necessary for HMW complex formation (Wolf et al. 2020). In our study, the HMW bands of *Cr*PGRL1 were absent in the C63SC167S and C284SC287S variants. This suggests that not only C63 and C167 but also C284 and C287 are involved in the formation of the HMW–PGRL1 complex; however, the role of each cysteine residue in complex formation is unclear.

### PGRL1 is unstable in C284 and C287 CS variants

In the PGRL1 stability assay, PGRL1 accumulation decreased under high light only in the C284SC287S and C287S variants (Fig. 4 and Figure S6). Cycloheximide significantly accelerated the decrease in PGRL1, indicating that the decrease in PGRL1 under high light was caused by protein degradation. There are 3 explanations for how CS substitutions of the C284, C287, or C284 and C287 affect PGRL1 stability: (1) disruption of the dimerization, (2) absence of a binding partner, and (3) disruption of coordination with a putative metal cofactor. Dimerization of PGRL1 may protect against proteolysis; however, the putative dimer was absent not only in C284SC287S but also in C63SC167S, exhibiting stable PGRL1 accumulation (Figure S4). In addition, PGRL1 appeared to be mostly monomeric under light. Thus, these results rule out possibility (1). We hypothesized that the binding of PGR5 to PGRL1 stabilizes PGRL1; however, we did not observe a significant decrease in PGRL1 levels in *pgr5* during the stability assay, suggesting that PGRL1 stability is PGR5 independent. According to Hertle et al., PGR5 level per chlorophyll was approximately one-eighth that of PGRL1 in *A. thaliana* (Hertle et al. 2013). If this also occurs in *C. reinhardtii,* PGR5 probably does not bind PGRL1 in a stoichiometric manner and does not significantly influence PGRL1 stability. Then, possibility (2) is unlikely. Residues C284 and C287, in addition to another cysteine pair of the C-terminal region (C256 and C259), were predicted to serve as zinc-binding sites (Chaturvedi et al. 2024; Walz et al. 2025). Mutations in ligands coordinating a metal cofactor, such as zinc or iron-sulfur cluster, may cause protein destabilization, as is commonly observed in metalloproteins (Tang et al. 2014; Xu and Moller 2011). Notably, under oxidative stress, where the ligand is readily oxidized, the metal cofactor can be released, leading to protein destabilization. Therefore, we conclude that possibility (3) is the most plausible explanation.

### Role of C284 and C287 in PGRL1 in PSI photoprotection

C284 and C287 are likely required for PGRL1 stabilization through metal cofactor binding. Additionally, C284 and C287 are likely to have a crucial role in the interaction with PGR5. The absence of PGRL1 led to the loss of PGR5 accumulation, presumably due to a lack of binding partner (DalCorso et al. 2008; Johnson et al. 2014). In addition, CS substitutions at the C-terminal region of PGRL1 induced a loss of PGR5 accumulation.

Four cysteine residues in the C-terminal region of PGRL1 were predicted to serve as ligands for a zinc atom (Chaturvedi et al. 2024; Walz et al. 2025); therefore, we used AlphaFold3 to predict the structure of mature *Cr*PGRL1 coordinating with a zinc atom and interacting with *Cr*PGR5 (Figure S9). Regarding the prediction accuracy, predicted local distance difference test (pLDDT) scores of the predicted structure were generally confident (>70), excluding the N-terminal region of *Cr*PGRL1 (Ser41–Ly73, Ile159–Lys172); a loop between transmembrane helices (Val211–Glu216); and the N-terminal region of *Cr*PGR5 (Lys53–Thr74) (shown in yellow-orange in Figure S9). Although *Cr*PGRL1 contains an extended α-helix domain at the C-terminus, the structure of the middle part of the C-terminal region, in which the peptide forms a β-sheets-rich domain and C256, C259, C284, and C287 coordinate the zinc atom, resembles that predicted in *A. thaliana* (Chaturvedi et al. 2024; Walz et al. 2025). Although C167, which is predicted to interact with Trx in *A. thaliana,* and 4 cysteine residues in the C-terminal region appear to be located closely (Chaturvedi et al. 2024; Figure S9), the pLDDT score for Cys167 was low (<50), indicating low confidence in this local region. Therefore, the theory that the C-terminal cysteine substitutions structurally affect the interaction between Trx, and PGRL1 dimerization should be interpreted with caution. In predicting the *Cr*PGRL1–*Cr*PGR5 interaction, template modeling score and interface predicted template modeling score were 0.65 and 0.67, respectively. These values indicate moderate overall confidence and suggest the presence of a protein–protein interface. *Cr*PGR5 appears to interact with PGRL1 near the C-terminal β-sheet-rich region containing C284 and C287 (Figure S9), implying that CS substitutions may alter the conformation and disrupt the PGRL1–PGR5 interaction. Although AlphaFold3 did not predict any major conformational changes after cysteine substitution in *Cr*PGRL1, our in vivo data showed that the C-terminal CS substitution impaired PGRL1 stability and disrupted PGR5 accumulation. Such discrepancies are presumably not uncommon, because AlphaFold predictions may not accurately reflect the conformational flexibility or environment-dependent structural responses of proteins (Tunyasuvunakool et al. 2021).

PGR5 possesses a cysteine residue that is predicted to interact with *At*PGRL1 (Hertle et al. 2013). In our structural prediction, the cysteine residue of *Cr*PGR5 did not interact directly with the cysteine residues of *Cr*PGRL1 (Figure S9). Therefore, the interaction between PGR5 and PGRL1 may occur independent of redox-mediated cysteine–cysteine interactions. According to Buchert et al. (2020), a substitution of the cysteine residue in PGR5 did not affect its function.

The PSI photosensitive phenotype observed in the C-terminal CS variants–C284SC287S, C284S, and C287S–is likely caused by the absence of PGR5, similar to the phenotype of *pgrl1* (Figs. 3a, 5 and Figure S7, B–D). PGR5 is predicted to function in ferredoxin-binding, FNR-binding, or participation in the Q-cycle (Munekage et al. 2002; DalCorso et al. 2008; Mosebach et al. 2017; Buchert et al. 2020). Therefore, anchoring PGR5 to the thylakoids through its interaction with PGRL1 is crucial for mediating PGR-dependent CEF, and consequently, for PSI photoprotection.

## Conclusion and perspectives

We demonstrated that residues C284 and C287 of PGRL1 are required for its stabilization under light stress and for PGR5 accumulation under growth conditions. These residues may contribute to PGRL1 dimerization and interaction with Trx, similar to residues C63 and C167. Thus, C284 and C287 play important roles in maintaining PGRL1 stability and enabling PGR5 accumulation, both of which are required for PGRL1-dependent PSI photoprotection. Further biochemical characterization would help elucidate the precise binding manner between PGR5 and PGRL1 and the cofactor of PGRL1, consequently clarifying the role of PGRL1 in PSI photoprotection in *C. reinhardtii*.

### Accession numbers

Sequence data from this article can be found in the GenBank/EMBL data libraries under accession numbers Cre07.g340200 (PGRL1) and Cre05.g242400 (PGR5).

## Supplementary Material

kiag164_Supplementary_Data

## Data Availability

All data supporting the findings of this study are available within the article and its supplementary materials.
