# Supplementary material for: Importance of cysteine residues in the C-terminal region of PGRL1 for PSI photoprotection in Chlamydomonas reinhardtii
Source: Plant Physiol. 2026 Mar 24;201(1):kiag164. doi: 10.1093/plphys/kiag164 (PMC13181399; doi:10.1093/plphys/kiag164)
Supplement: kiag164_Supplementary_Data [file kiag164_supplementary_data.pdf]

Supplementary Figures and Tables

Importance of cysteine residues in the C-terminal region of PGRL1 for PSI photoprotection in *Chlamydomonas reinhardtii*

Takahashi et al.

Supplemental Figures S1−S6.
Supplemental Tables S1 and S2.

Supplementary Figure S1

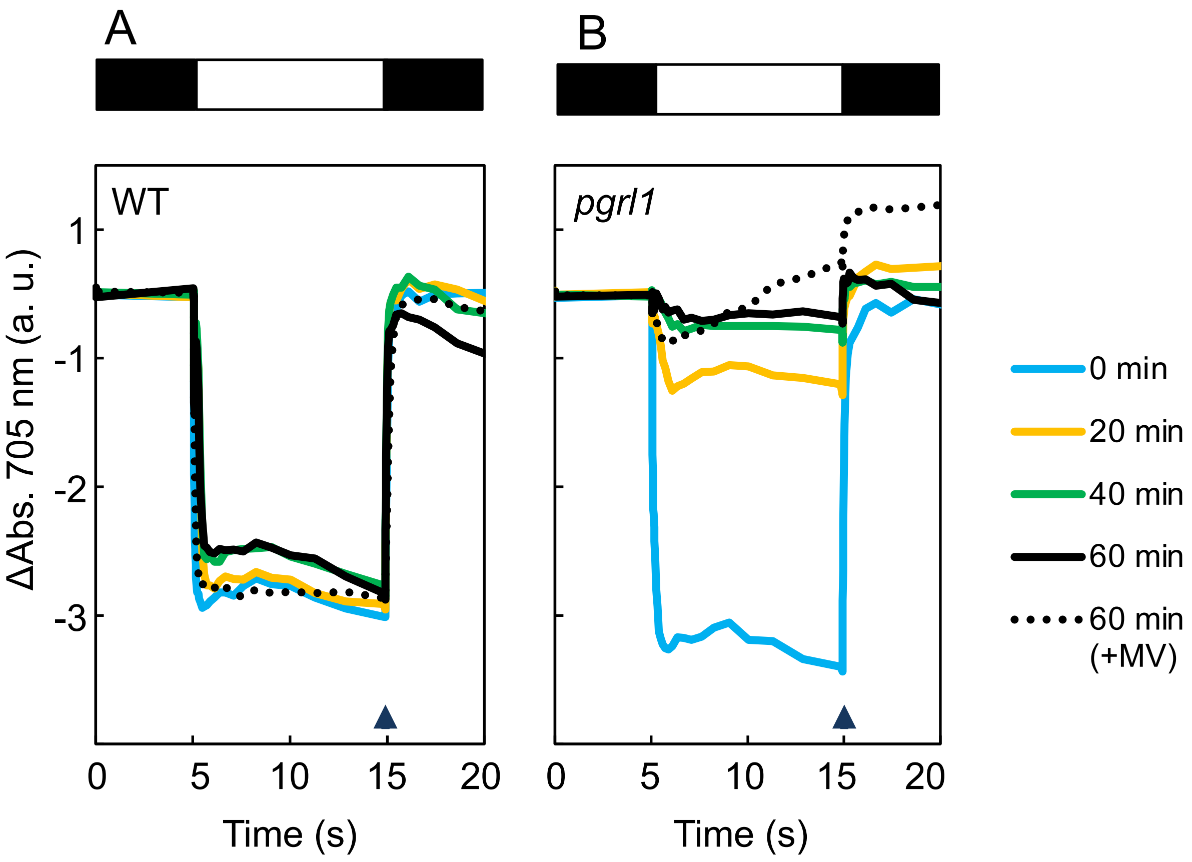

Supplemental Figure S1. P700 photooxidation kinetics in WT and *pgrl1* after high-light illumination. P700 photooxidation was measured in WT (A) and *pgrl1* (B). The measurement was performed in the presence of 10 µM DCMU under actinic light at 940 µmol photons m^{-2} s^{-1} for 10 seconds, represented by the white bar above the kinetics. Pulse illumination was applied at the end of the actinic light, indicated as black arrowheads. +MV represents measurement in the presence of 1 mM methylviologen (MV).

Supplementary Figure S2

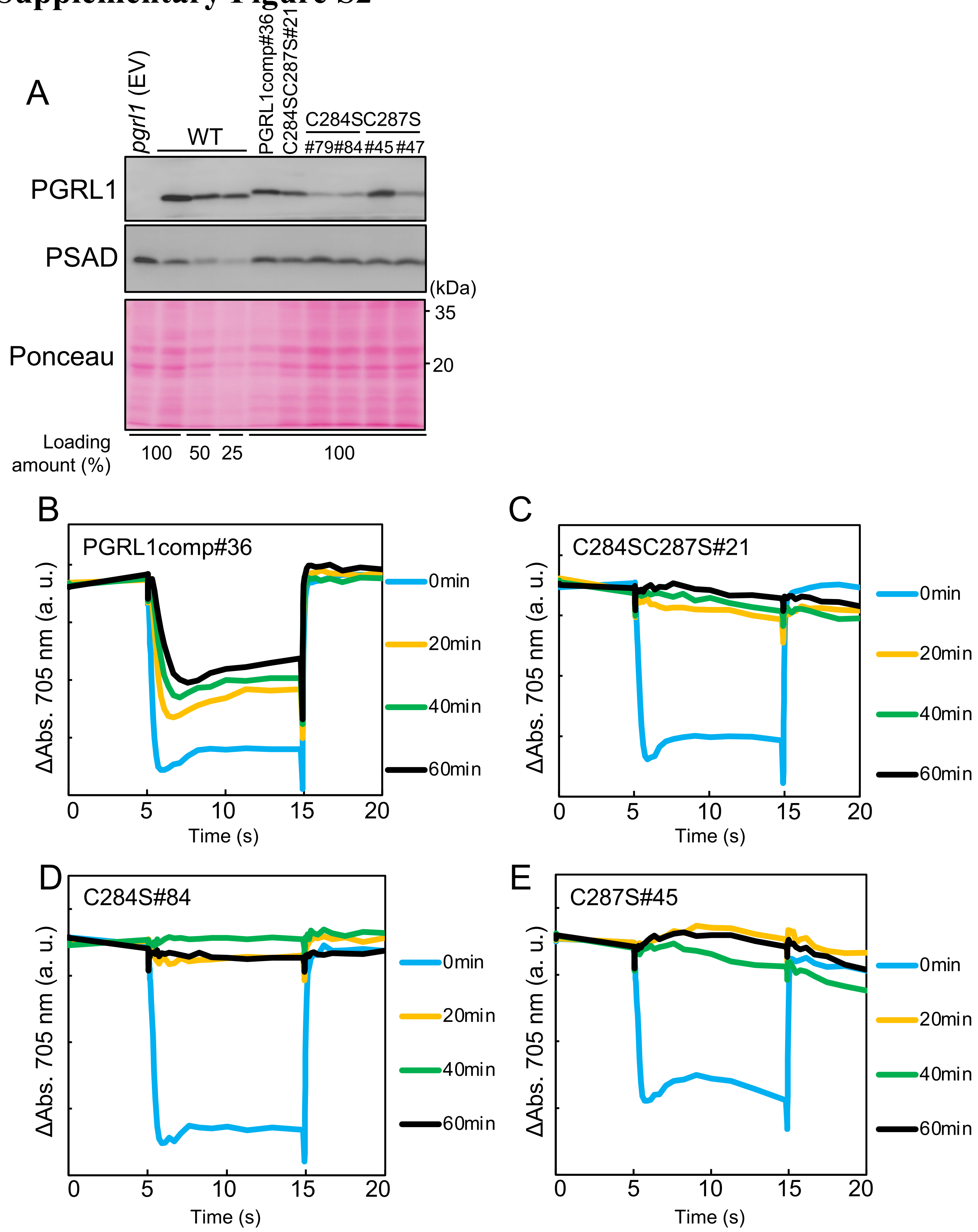

Supplemental Figure S2. PSAD protein level and P700 photooxidation kinetics in the C-terminal CS variants. A) Total cellular proteins (equivalent to 1 μg Chl as 100%) extracted from WT, *pgrl1*, PGRL1comp, C284SC287S, C284S, and C287S strains grown under 80 μmol photons m^{-2} s^{-1} were subjected to immunoblotting analysis using PSAD antiserum. Ponceau-stained protein profiles are shown as a loading control. PSAD was detected in the same blot shown in Fig. 2B, lower panel. B-E) kinetics of P700 photooxidation in the PGRL1comp#36, represented as 2#36 (B), the C284SC287S variant, as 7#21 (C), C284S, as 9#84 (D) and C287S, as 10#45 (E). The measurement was performed in the presence of 10 μM DCMU under the actinic light at 150 μmol photons m^{-2} s^{-1} for 10 seconds. The measurement was done at 0, 20, 40 and 60 min during the high-light illumination for 1 hour.

Supplementary Figure S3

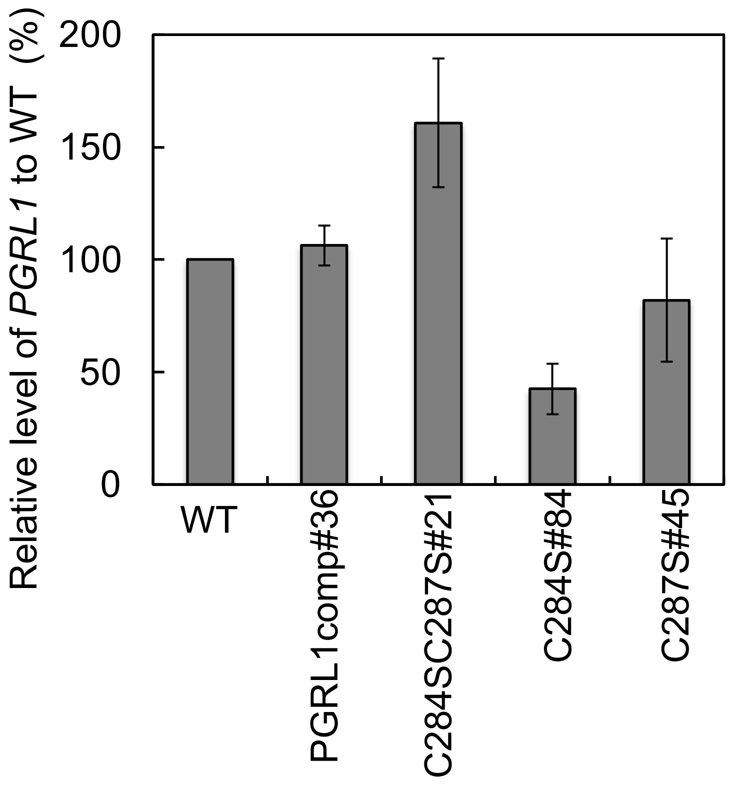

Supplemental Figure S3. *PGRL1* transcript level in the C-terminal CS variants.
The ratio of *PGRL1* per *CBLP* in WT, PGRL1comp#36, the C284SC287S, C284S, and C287S variants was quantified by RT-qPCR. The relative levels of *PGRL1*/*CBLP* compared to WT are shown in the graph. Values represent means of three biological replicates, and error bars indicate ± S.D.

Supplementary Figure S4

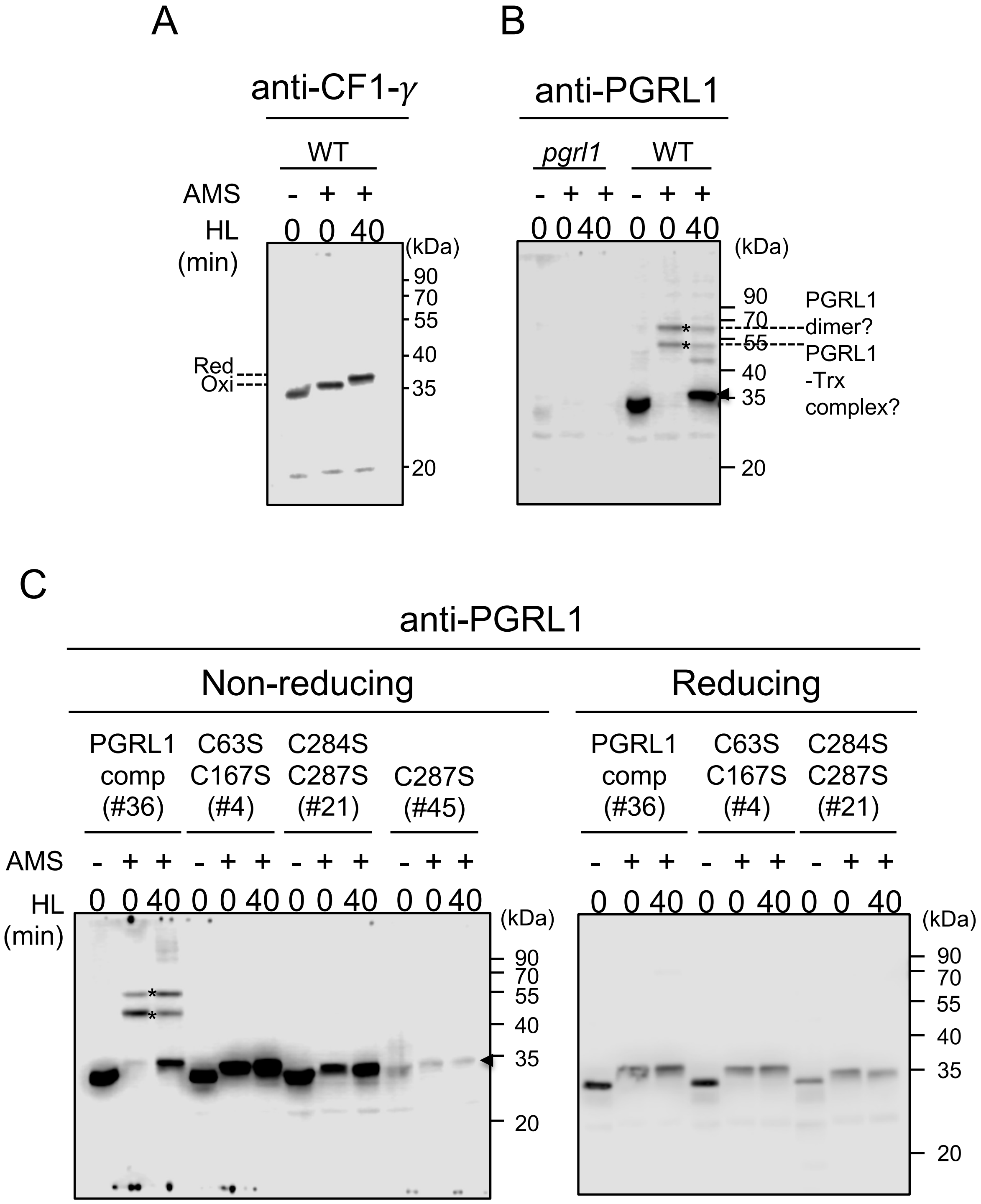

Supplemental Figure S4. Redox assay of PGRL1 protein.
Free thiol group-labeled proteins in the total cellular proteins were analyzed by immunoblotting. A) Photo-reduction pattern of ATP synthase CF1-γ subunit in WT is shown as a positive control of the assay. 10 µg of protein was loaded in each lane. B) The photo-reduction pattern of PGRL1 in *pgrl1* and WT is shown. 15 µg of protein was loaded in each lane. C) The labeled proteins extracted from the PGRL1comp#36, C63SC167S, and C284SC287S variants were migrated by non-reducing SDS-PAGE (right panel) and by reducing SDS-PAGE (left panel) and subjected to immunoblotting. 7.5 µg of protein for the complemented strain (#36) and C63SC167S, 15 µg of protein for C287SC284S, and 10 µg of protein for C287S were loaded in each lane, respectively.

Supplementary Figure S5

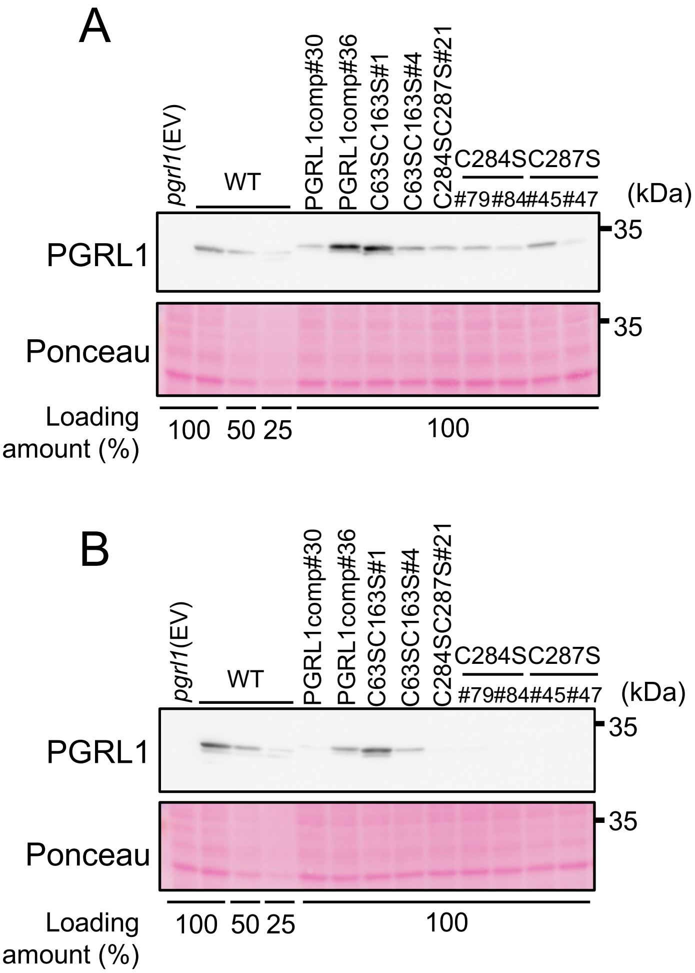

Supplemental Figure S5. Comparison of PGRL1 accumulation between mixotrophically and photoautotrophically grown cells.

Total cellular proteins (equivalent to 1 μg Chl as 100%) extracted from WT, *pgrl1*, PGRL1comp strains, C284SC287S, C284S, and C287S variants grown under mixotrophic (A) and photoautotrophic condition without CO_2 supply (B) were subjected to immunoblotting analysis using PGRL1 antiserum. Ponceau-stained protein profiles are shown as a loading control.

Supplementary Figure S6

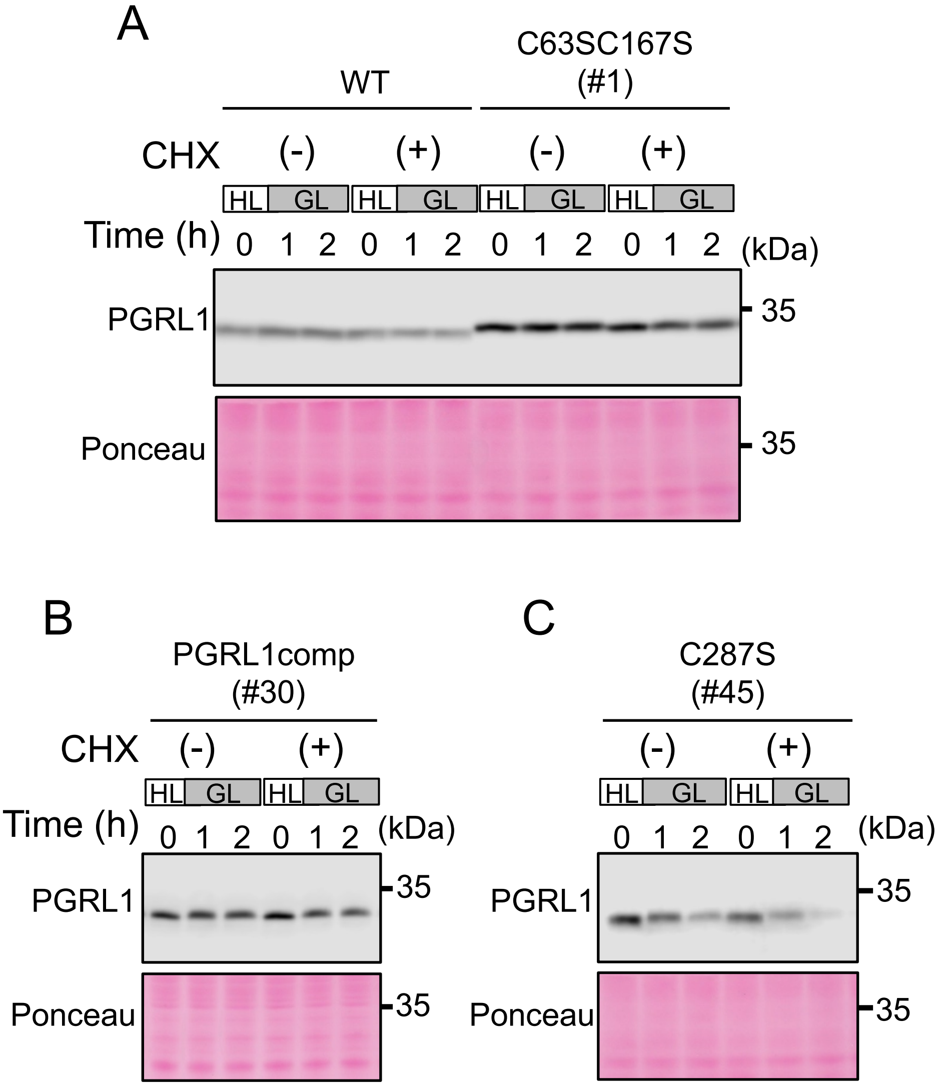

Supplemental Figure S6. Stability of PGRL1 in WT, the PGRL1comp, C63SC167S, and C287S strains. PGRL1 level after high-light treatment in the absence or presence of 10 μg ml^{-1} cycloheximide (CHX) in WT and C63SC167S#1 (A), PGRL1comp#30, in which PGRL1 accumulated less than that in WT (B), and C287S#45 (C). The experiments were performed as shown in Fig. 4.

Supplementary Figure S7

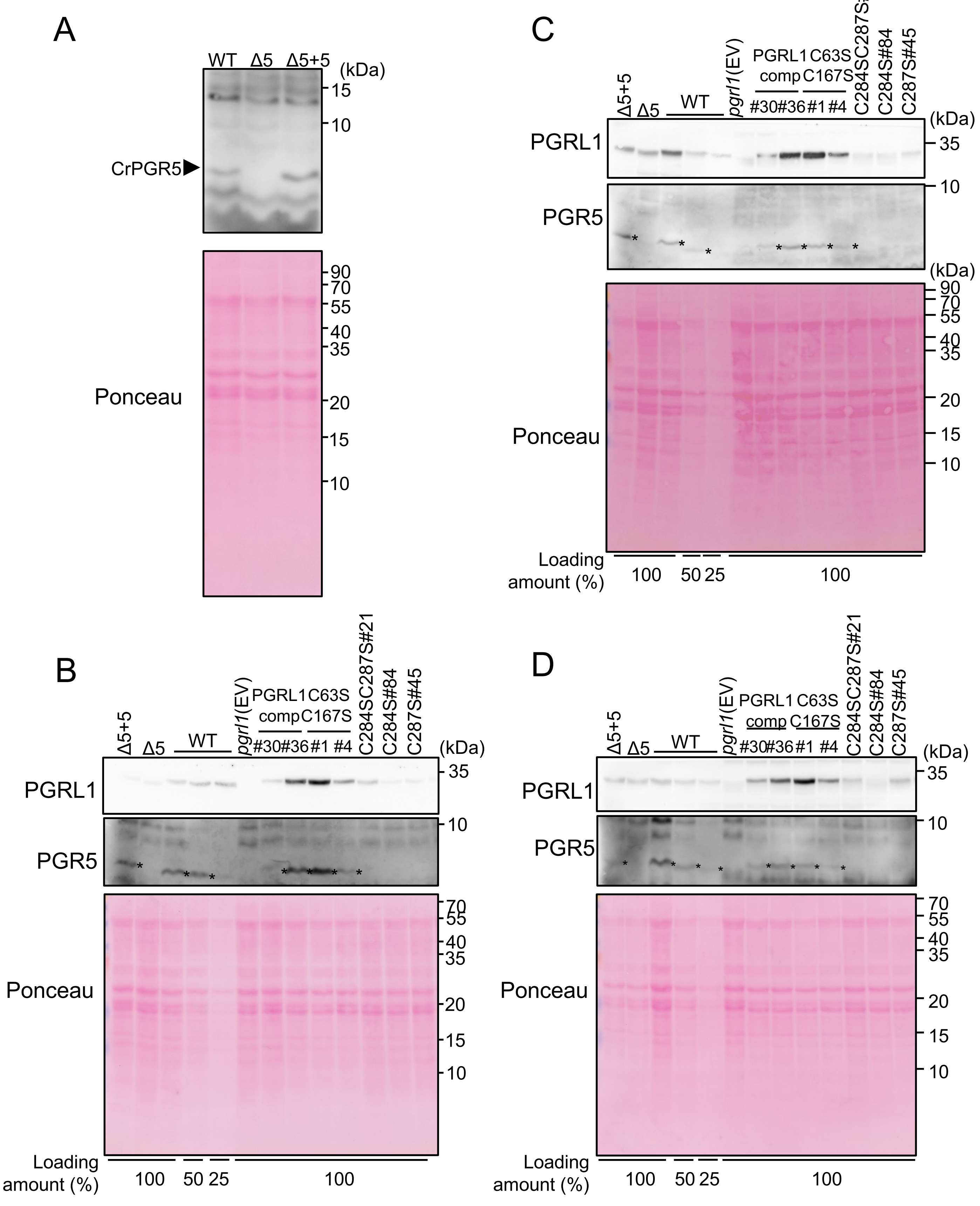

Supplemental Figure S7. Biological replicates of PGR5 level in the PGRL1 CS variants.
A) To test the antiserum against CrPGR5, total cellular proteins (equivalent to 1 µg Chl as 100%) extracted from T222+ (WT), *pgr5* (Δ5), and *pgr5*+PGR5 (Δ5+5) were subjected to immunoblotting. The black arrowhead indicates PGR5 band. B-D) Two biological replicates were analyzed as performed in Fig. 5. The asterisks indicate the position of the PGR5 band.

Supplementary Figure S8

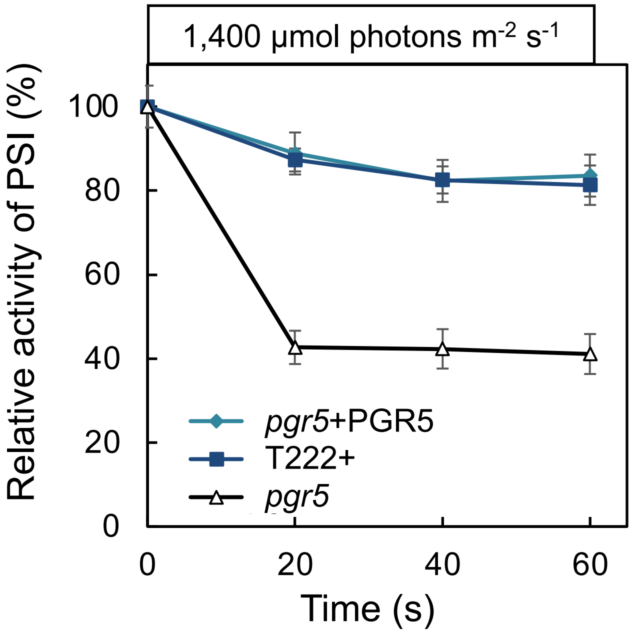

Supplemental Figure S8. PSI activity in *pgr5* under high light-conditions.
The activity of PSI in WT (T222+), which is the recipient strain of *pgr5*, *pgr5*, and *pgr5*+PGR5 under high light conditions, was determined as performed in Fig. 1C. Filled diamond, filled square, and open triangle represent *pgr5*+PGR5, T222+, and *pgr5*, respectively. Values represent means of three biological replicates, and error bars indicate ± S.D.

Supplementary Figure S9

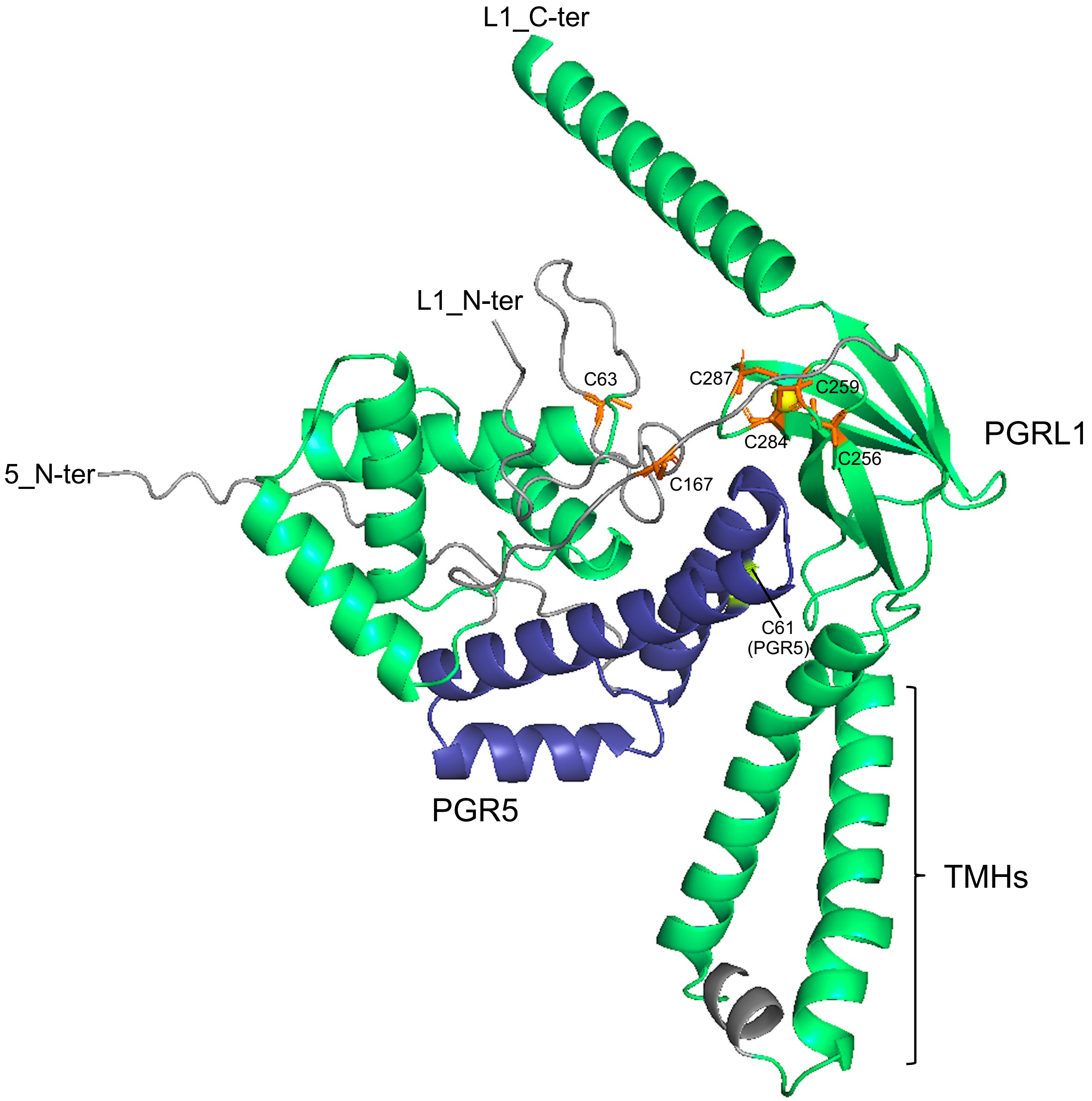

Supplemental Figure S9. Structural model of CrPGRL1 coordinating a zinc atom and interacting with CrPGR5, predicted by AlphaFold3.

The mature protein sequences of CrPGRL1 (with zinc coordination) and CrPGR5 from *Chlamydomonas reinhardtii* were used as input for structure prediction with AlphaFold3. The predicted model was further modified using PyMOL. CrPGRL1 and CrPGR5 are shown in lime green and deep blue, respectively. Regions with low predicted local distance difference test scores (< 50) are shown in gray. The cysteine residues in CrPGRL1 and CrPGR5 are highlighted in orange and limon yellow, respectively. The zinc atom is shown as a yellow sphere. L1-N-ter, TMHs, L1-C-ter, and 5-N-ter indicate the PGRL1-N-terminus, its transmembrane helices (as previously predicted by DalCorso et al., 2008, *Cell*), its C-terminus, and the PGR5-N-terminus, respectively. In predicting the *Cr*PGRL1–*Cr*PGR5 interaction, template modelling score and the interface predicted template modelling score are 0.65 and 0.67, respectively.

Supplemental Table 1

The number of screened and isolated PGRL1 CS-substituted clones

Introduced sequence to *pgrl1*	Screened by immunoblotting	Accumulating PGRL1 at 20–30% of that in WT	Accumulating PGRL1 at 60–150% of that in WT
C63SC167S	5	0	4
C256SC259S	218	0	0
C256S	56	0	0
C259S	56	0	0
C284SC287S	32	2	1
C284S	85	5	0
C287S	64	4	1

Supplemental Table 2

The primers used in this study

Primer name	Sequence
CrPGRL1OHpET21_Fw	5'-GGACAGCAAATGGGTCGGATGCAGACCACCGTGCTC-3'
CrPGRL1OHpET21_Rv	5'-GTGGTGGTGGTGGTGGTGCGCAGCGGCCTTAGCCTT-3'
CrPGRL1cdsOHpSXY2007_Fw	5'-CTACTCACAACAAGCCCAATGCAGACCACCGTGCTC-3'
CrPGRL1cdsOHpSXY2007_Rv	5'-CGGTCCAGCTGCTGCCATTACGCAGCGGCCTTAGC-3'
proPSAD	5'-CGAGCAAGCCAGGGTTAG-3'
terPSAD	5'-CCGAGCTCCGATCCCG-3'
PGRL1rtPCR_Fw	5’-GGTGGTGGCTGAGACGTC-3’
PGRL1rtPCR_Rv	5’-TTACGCAGCGGCCTTAGCC-3’
CBLPrtPCR_Fw*	5’-GAGTCCAACTACGGCTACGCC-3’
CBLPrtPCR_Rv*	5’-CTCGCCAATGGTGTACTTGCAC-3’

* The CBLP-specific primers were described in Tokutsu et al., (2019) *Scientific Report.* 9:2820.
